# *In vitro* investigation of the ruminal digestion kinetics of different nitrogen fractions of ^15^N-labelled timothy forage

**DOI:** 10.1371/journal.pone.0203385

**Published:** 2018-09-17

**Authors:** M. Vaga, P. Huhtanen

**Affiliations:** Department of Agricultural Research for Northern Sweden, Swedish University of Agricultural Sciences, Umeå, Sweden; The University of Sydney, AUSTRALIA

## Abstract

An *in vitro* method based on ^15^N-labelled forage nitrogen (N) was developed to study ruminal N metabolism of soluble N (SN), insoluble N (ISN) and neutral detergent insoluble N (NDIN) fractions of timothy forage. Timothy grass was grown on replicated experimental plots with one plot receiving ^15^N-labelled and the other unlabelled N fertilizer. Harvested grass was preserved as dried grass or as formic acid treated or untreated silage. The intact forages and their corresponding N fractions were incubated in buffered rumen fluid *in vitro* to determine degradation parameters based on the ^15^N fluxes between labelled feed N and ammonia N pools. A high percentage (25–38%) of ^15^N-labelled ammonia disappeared from ammonia N pool during the first 15 min of incubation. Microbial uptake of dried grass SN fraction was higher than of silage SN fractions. Fractional degradation rates of SN from formic acid treated silage, untreated silage and dried grass during the first 6 hours of incubation were 0.145, 0.125 and 0.115 /h, respectively. By the end of the incubation period (28 h), 69, 66 and 43%, of the SN fraction of formic acid treated silage, untreated silage and dried grass, respectively were recovered as ammonia. The percentage of ISN fractions degraded to ammonia N were 9, 34 and 27%, respectively. Based on the changes in ^15^N-labelled ammonia N pool in blank incubation and appearance of ^15^N to ammonia N pool from ^15^N-labelled NDIN fractions, it was estimated that a significant portion of microbial lysis occurred when incubations were carried out for longer than 20 hours. With dried grass the contribution of ammonia N for microbial N synthesis was greater than with silages. Use of ^15^N-labelled forages together with this *in vitro* method is a promising technique for determining soluble N degradation parameters, but it requires further development to be used for determining degradation parameters of insoluble N fractions and work with whole feeds.

## Introduction

Grass forage, and especially ensiled grass is the major component of dairy cow diet in Northern Europe, often consisting >50% of diet DM. Therefore even small improvements in forage protein utilisation could reduce the need for supplementary protein [1]. However, the processes during silage fermentation change the composition of forage, particularly the content of readily fermentable components such as soluble proteins and carbohydrates [2]. Similarly to ruminal fermentation, microbes in silage use water-soluble carbohydrates during silage fermentation to produce energy for growth. This leads to losses of energy and changes in the composition of the fermentable substrate available for ruminal organisms [3,4], and consequently reduced ruminal microbial efficiency, especially in poorly preserved forages [3]. In studies with growing [5] and lactating cattle [6], ruminal protein degradability was lower with hay-based diets compared with silage-based diets. Jaakkola and Huhtanen [5] reported greater microbial nitrogen (**N**) flow from the rumen with silage-based than with hay based diets, but the differences in total non-ammonia N flow were not significant. These conflicting results indicate a need to improve understanding of the effects of forage preservation method on ruminal protein metabolism in dairy cows as total rumen protein degradability alone seems insufficient for evaluating forage protein value. Restricted silage fermentation has been shown to increase production of milk fat and protein, mainly due to increased dry matter intake [7].

The models employed to optimise nutrient efficiency within existing feeding evaluation systems such as CNCPS [8], NRC [9] and Volden [10] use different levels of N fractionation. Furthermore, changes in the fractional composition of plant N due to preservation method (ensiling, drying, etc.) can affect ruminal N metabolism and the supply of metabolisable protein to the small intestine [11]. Most current feed evaluation systems rely on protein degradation parameters estimated by the *in situ* method [12]. However, the inherent shortcomings of the *in situ* method, in which feedstuff is incubated inside porous nylon bags inside cows rumen to measure disappearance (degradation) of substrate over period of time, make it difficult to accurately evaluate all N fractions [13]. The escape of soluble N from nylon bags, restricted microbial access to feed in the bags or microbial contamination of feed residues are likely, but not only causes of the low reproducibility of the *in situ* method [14]. Therefore various *in vitro* systems have been developed to estimate ruminal protein degradation characteristics [15,16]. Techniques involving ^15^N-labelled forage have made it possible to investigate N kinetics in the rumen [17] and the nitrogen isotope ^15^N has been successfully used as a microbial N marker in *in vitro* studies [18,19]. When used together with nitrogen isotope ^15^N to measure degradation of crude protein (**CP**) and/or microbial N synthesis, this allows ruminal protein metabolism to be better predicted [18]. The objectives of the present study were therefore to: 1) develop an *in vitro* method using ^15^N-labelled forages to estimate the *in vitro* ruminal digestion kinetics of plant soluble N (**SN**), insoluble N (**ISN**) and neutral-detergent insoluble N (**NDIN**) fractions of dried and ensiled timothy grass, and 2) compare the effects of preservation methods on the composition of N fractions and digestion kinetics of timothy grass.

## Material and methods

### Production of ^15^N-labelled forages

Timothy (*Phleum pratense*, cv. Grindstad) was grown in two replicate plots of 2 m^2^ each in the experimental field at Röbäcksdalen research station, Umeå, Sweden (63°35´N, 20°45´E). Both plots received 10 g N/m^2^ as NH_4_NO_3_ fertiliser (applied in water solution) and 1.8 g/m^2^ of phosphorus (**P**) and 9.5 g/m^2^ of potassium (**K**) as commercial compound fertiliser on 8 May 2013, when approximately 5 cm shoots were visible. To produce ^15^N labelled forages with 1–2 atom% excess of ^15^N, the timothy grass was fertilised with labelled ^15^NH_4_^15^NO_3_ (Larodan Fine Chemicals AB, Malmö, Sweden) containing 2% ^15^N/N. Control timothy grass received unlabelled commercial NH_4_NO_3_.

Forages were harvested on 9 June 2013 when grass was between boot and early flowering stage. One-third of the forage from each plot was dried (dried grass) in a forced air oven for 48 h at 50°C to avoid protein denaturation. The aim was to compare traditional hay against silages, and therefore oven drying was used to avoid quality changes due to bad weather conditions during the harvest period. The rest of the forage was chopped using handheld gardening shears and thereafter wilted for 2 h on a paper sheet outdoors. The wilted forage was conserved in 1-L glass jars without additives (untreated silage) or with formic-acid based additive (formic acid treated silage) (ProMyr XR801, Perstorp AB, Perstorp, Sweden) applied at a rate of 4 mL/kg wilted forage. After 100 days, the silages were removed from the jars, packed into polythene grip-seal bags and kept frozen at -20°C until freeze-dried or analysed. Dry matter concentration of preserved forages and N fractions was determined by drying at 105°C for 16 h and ash concentration by incinerating at 500°C for 4 h. All forages and ISN and NDIN fractions were analyzed for CP [20] using a 2020 Digestor and a 2400 Kjeltec Analyzer Unit (Foss Analytical A/S, Hilleröd, Denmark). Metabolizable energy concentration in silages and dried grass was calculated following the procedure of Lindgren [21]. The frozen silage samples were thawed and pressed, and the pH in the liquid was measured with a pH meter (Metrohm, Herisau, Switzerland) and further analyzed for VFA and lactic acid according to Ericson and André [22]. Analysis for silage ammonium-N was done by direct distillation after adding MgO with Kjeltec 2100 Distillation Unit (Foss Analytical A/S). Organic matter digestibility was determined for all forages after 96 hr gas *in vitro* incubations, as described by Hetta et al., [23]

### Preparation of forage N fractions

**Fig 1:**
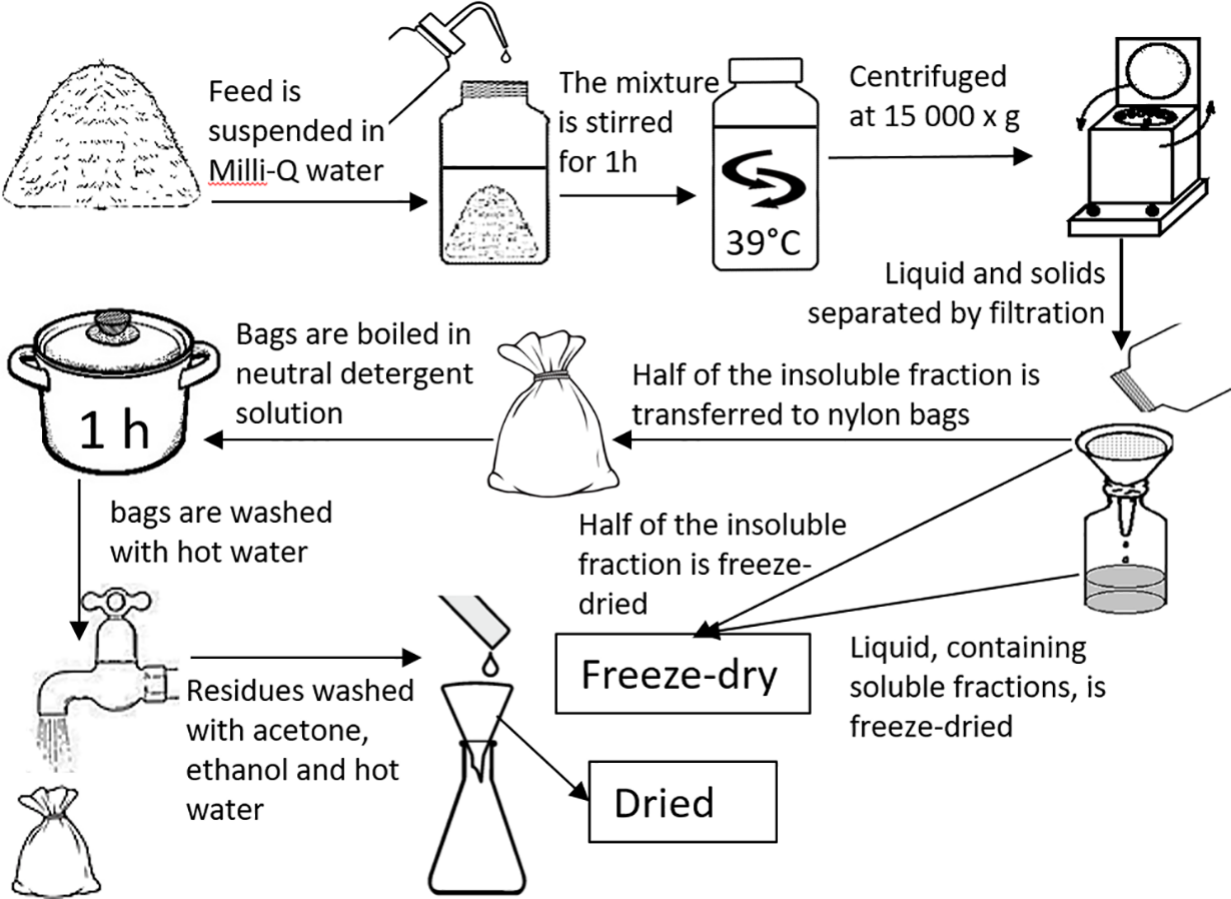
Procedures used for extracting forage N fractions from silages and dried grass. (Images acquired from http://getdrawings.com/ and procedures notes DOC316.53.01186, Hack company/Hach Lange GmbH).

Freeze-dried silage and oven-dried grass samples were milled through a 1 mm screen with a Cyclotec 1093 sample mill (Foss Tecator, Högnäs, Sweden) and then separated into the three N fractions: SN, ISN and NDIN. One-quarter of the milled sample material was retained as a whole forage. Fig 1 depicts the procedures of N fractionation. Portions of 20 g were suspended in 400 mL of ultrapure water (**Milli-Q**, Merck Millipore Corporation, Darmstadt, Germany) and stirred for 1 h at 39°C. The suspension was centrifuged at 15 000 x g for 15 min (Avanti J26S XP, Beckman Coulter, Inc. Brea, CA, USA) and filtered through Whatman no. 1 filter paper. The filtrate consisting of soluble fraction was frozen at -20°C and freeze-dried. The N content in soluble fraction was determined simultaneously with determination of ^15^N enrichment with Flash EA 2000 elemental analyser (Thermo Fisher Scientific, Bremen, Germany) as detailed under section “Determination of ^15^N abundance”. The insoluble residues were rinsed twice with milli-Q water to remove all remaining soluble N and half were frozen at -20°C and later freeze-dried (ISN fraction). The remaining half was allocated into nylon bags and then boiled in neutral detergent solution (without sodium sulphite) for 1 h to prepare the NDIN fraction. After extraction, the bags were thoroughly rinsed in hot running water and dried at 60°C overnight. The dried residues were washed several times with boiling water, ethanol and acetone to remove all neutral detergent residues.

Neutral detergent fibre (**NDF**) was extracted from a good quality timothy grass silage using the same procedure as for NDIN fraction, but with added Na_2_SO_3_ (0.25 g per 1 g sample) to remove NDF-bound N.

### Experimental design

The three N fractions were incubated in rumen fluid *in vitro* as mixed substrates. These substrates were prepared from the respective N fraction, isolated NDF, soybean meal and a carbohydrate mixture containing pectin, starch and maltose (2:1:1 on DM basis) to achieve similar concentrations of N and NDF as in the whole forages (Table 1). Isolated NDF was mixed with the SN fraction but ISN and NDIN fractions were supplemented with soybean meal and carbohydrate mixture dissolved in buffer solution [24]. The experimental design included two treatments (non-labelled and ^15^N-labelled), three forages (formic acid treated and untreated silage and dried grass) and four fractions (whole forages, SN, ISN and NDIN). A complete randomised design was used with the rule that each substrate had to be represented in each run at least once. Each forage and the substrates containing the N fractions were incubated in three *in vitro* runs with the incubation bottle being an experimental unit. In each *in vitro* run one sample of each substrate was represented and one-third of the substrates was replicated, resulting in four replicate observations per sample. All samples within a run were randomly distributed spatially within and between water baths. Within each run, a blank (buffered rumen fluid without a sample) and a blank with ^15^N-labelled ammonium in the buffer were incubated in duplicate.

**Table 1:** In *vitro* substrate formulation (mg/g DM) and chemical composition.

			Substrates^1^		
	Whole forage		SN	ISN	NDIN
Forage		1000	0	0	0
SN		0	330–562^2^	0	0
ISN		0	0	670–684	0
NDIN		0	0	0	507–536
Soybean meal		0	0–198	109–212	163–262
NDF		0	438–480	0	0
CHO^3^		0	0	111–205	196–300
Chemical composition					
DM, g/kg		860–940	936–950	940–950	940–950
CP, g/kg DM		132–166	142–168	147–159	143–155
NDF, g/kg DM		500–550	480–546	500–547	500–547

^1^ Substrates containing: SN–soluble N fraction; ISN–insoluble N fraction; NDIN–neutral detergent-insoluble N fraction.

^2^ The substrates were prepared to have similar CP and NDF concentrations. Since concentrations of CP and/or NDF in the same fraction differed between forages, the ratios of substrate components varied.

^3^ CHO–carbohydrate mixture containing pectin, starch and maltose (2:1:1 on DM basis).

### In vitro procedures

All animals used for *in vitro* procedures were treated and kept with permission acquired beforehand from the Swedish Ethical Committee on Animal Research represented by the Court of Appeal for Northern Norrland in Umeå, Sweden and with permission from ethical committee of Umeå Djurförsöksetiska nämnd (Document nr. A97-07). Rumen fluid was collected between the morning milking and morning feeding from two rumen-cannulated lactating Nordic Red cows fed a diet containing grass silage and commercial concentrate (0.60:0.40 on DM basis) twice a day. A total of 800 mL of rumen fluid was strained through four layers of cheesecloth into Erlenmeyer flasks: Rumen fluid together with 8 g of a mixture of carbohydrates (maltose, starch, xylose, pectin; 2:1:1:1) and 2.8 g of NaHCO_3_) was pre-incubated for 3 hours in a water bath at 39°C. The purpose of pre-incubation [25] was to reduce the amount of digestible substrate originating from the rumen, by mechanically removing larger particles and giving time for microbes to degrade the rest. All procedures with rumen fluid were performed under a constant flow of CO_2_ to maintain anaerobic conditions. The rumen fluid mixture was stirred for 5 min and then the stirrer was turned off. After 25 min, the buoyant layer on rumen fluid was removed by vacuum aspiration. Thereafter the stirrer was turned on again and the rumen fluid mixture was continuously stirred for 2.5 h. The pre-incubated rumen fluid was mixed into buffered mineral solution [24] with a 1:4 volume ratio of rumen fluid to buffer. Substrates (forages and N fractions) of 500 mg were incubated in 60 mL of buffered rumen fluid in 250 mL serum bottles (Schott, Mainz, Germany). The freeze-dried forage SN fraction was solubilised in 4 mL of buffer solution (same as used for rumen fluid) before being added to the incubation bottles. Same amount of buffer solution was added to the other incubation bottles. In bottles containing unlabelled substrates, the ammonia in the inoculum was labelled with ^15^N by adding 1 mg of enriched (^15^NH_4_)_2_SO_4_ with 99% ^15^N/N (Larodan Fine Chemicals AB, Malmö, Sweden) dissolved in 0.2 mL of milliQ water. Bottles containing ^15^N labelled substrates received the same amount of non-enriched (NH_4_)_2_SO_4_. All bottles were kept in a water bath at 39°C for 44 h and continuously agitated.

The *in vitro* procedures were performed with a fully automated system [26] recording gas production every 12 min as described in detail by Hetta *et al*. [23]. Simultaneously with gas recordings, 0.6 mL of fluid from rumen samples was extracted at 0.25, 0.5, 1, 2, 3, 4, 6, 9, 20 and 28 h for determination of ammonia N and soluble non-ammonia N (**SNAN**) concentration in the liquid phase. The system for allowing collection of fluid samples during incubation is described in detail by Karlsson *et al*. [27]. The fluid samples were transferred to Eppendorf tubes containing 0.024 mL of 18 M H_2_SO_4_ for preservation and then frozen (-20°C). Prior to analysis, the samples were thawed and centrifuged at 12500 × g for 10 min. Thereafter 0.1 mL aliquots of supernatant were transferred to test-tubes and diluted to 1:20 with milli-Q water. The concentration of NH_3_-N was analysed using a continuous flow analyser (AutoAnalyzer 3 HR, SEAL Analytical Ltd) following the instructions provided by the manufacturer (Method No. G-102-93 Rev 7 (multitest MT7)).

### Determination of ^15^N abundance

To determine the ^15^N-atom% in forages and N fractions, the milled samples were further homogenised in a ball mill and a sample containing 20–100 μg N was weighed and closed within a tin capsule. The ^15^N-atom% was determined using a Flash EA 2000 elemental analyser (Thermo Fisher Scientific, Bremen, Germany) connected to a DeltaV isotope ratio mass spectrometer (Thermo Fisher Scientific, Bremen, Germany).

To determine the ^15^N-atom% for ammonia N, the frozen samples were thawed at room temperature and centrifuged at 12 000 x g for 5 min. Then 0.3 mL of supernatant was pipetted into a 2 mL micro centrifuge tube and 0.3 mL of 10 N NaOH was added. A polyethylene mesh was rolled up and placed within the mouth of the micro centrifuge tube. A fibreglass filter (⌀ 5.5 mm, Whatman GD/C) disc impregnated with 1 μL of 5 M H_2_SO_4_ was placed on top of the mesh and the cap was closed tightly. The tubes were kept for 4 days at room temperature under constant slow agitation on a rotary shaker. Thereafter, the filter discs were removed from the tubes and placed in a 96-well microtiter plate which was placed inside a desiccator together with a container of 18 M H_2_SO_4_ and dried overnight. The dry filter discs were sealed in tin capsules and sent for analysis. In addition, some untreated and acid-impregnated filter papers were prepared and added as controls to check for contamination.

To determine the N concentration and ^15^N-atom% in SNAN, the samples were thawed and centrifuged in the same way as the ammonia N samples. Each fibreglass filter disc was placed inside a tin capsule in a 96-well microtiter plate and 80 μL of supernatant were pipetted onto the filter paper. The plate was placed in a desiccator together with a container of 18 M H_2_SO_4_, and dried at 60°C overnight. On the following day, the samples were left to cool to room temperature within the desiccator, after which the tin capsules were sealed and placed back in the microtiter plate. The capsules were analysed for ^15^N-atom% the same way as the forages described before. The size of the SNAN pool in digesta was calculated by subtracting the ammonia N pool from the total SN pool.

### Calculations

The pool sizes of ammonia N and SNAN in the incubation bottles were calculated using the N concentrations determined in the respective samples and the volume of the total incubation medium. The change in volume of the incubation medium during the sampling period was taken into account.

The ^15^N-atom% values in unlabelled forages were very similar, and therefore an average of 0.372% [SD = 0.0014) was used to calculate ^15^N-atom% excess in labelled feeds. The ^15^N-atom% value measured in ammonia N and SN samples of unlabelled blanks were used as background ^15^N concentrations to calculate ^15^N atom% excess in the respective pools.

The size of the ^15^N excess pool (^**15**^**NEP**, μg) of the ammonia N pool (μg) was calculated as:

$${}^{15}NEP=Ammonia\;N\;pool\times {}^{15}N\;atom\%\;excess\;in\;rumen\;ammonia\;N/100$$ (1)

The fractional degradation rate of SN was calculated based on ^15^N disappearance from the SN pool, estimated from the slope of the linear regression line using natural logarithmic values of the ^15^NEP size.

Gas recordings were used to only check the normality of *in vitro* degradation processes. A mean asymptotic cumulative gas production (mL) was calculated per sample OM (g).

### Statistical analysis

The effects of preservation method on *in vitro* protein degradation parameters such as ammonia N concentrations and microbial uptake and fractional degradation rate of SN fractions was analysed separately for each feed using the mixed procedure in SAS (SAS Inst. 2008. Version 9.4 Inc., Cary, NC) and the following model:

$$Y_{ij}=\mu +T_{i}+r_{j}+e_{ij}$$ (2)

where μ is the overall mean, T_i_ is the treatment method (formic acid treated silage, untreated silage and dried grass), r_j_ is the random effect of run and e_ij_ is the error. Within ^15^N labelled and unlabelled forages and ISN and SN fractions the treatments were analysed for treatment × time interactions using following model:

$$Y_{ij}=\mu +T_{i}+P_{j}+TP_{ij}+r_{k}+e_{ijk}$$ (3)

where μ is the overall mean, T_i_ is the treatment method (formic acid treated silage, untreated silage and dried grass), P_j_ is time, TP_ij_ is the treatment × time interaction, r_k_ is the random effect of run and e_ijk_ is the error. Time was set as repeated measurement. Treatment effects and interactions were considered significant at *P* ≤ 0.05.

### Model development

The kinetic parameters describing degradation of forage N fractions were estimated using observed values of ^15^NEP. As the aim was to develop models to describe protein degradation *in vitro*, the estimations were made using only mean data values of 4 replicates, for each time point, and therefore it was not possible to perform further statistical evaluation of model predictions between treatments. The model describing SN degradation consisted of three compartments: ammonia N, SNAN and microbial N, where SNAN can be degraded to ammonia N or incorporated directly to microbial N. The model describing microbial N synthesis from ammonia N for SN and ISN had two compartments: ammonia N, and microbial N together with SNAN, where ammonia N can only be incorporated to microbial N. Estimated parameters in the models were defined to be significantly different from zero (*P* < 0.05), i.e. the fractional standard deviation (FSD = SD / mean) was below 0.5 for all parameters in all datasets. Only data from labelled ammonia N with unlabelled ISN fractions were used for the ISN model, because the appearance of ^15^N in ammonia and SNAN pools from labelled ISN fractions was too low to estimate the parameters satisfactorily. The models were developed and evaluated using WinSAAM version 3.0.7 software (www.winsaam.com; New Bolton Center, Biostatistics Unit, University of Pennsylvania) [28].

## Results

### Descriptive data on timothy silage and dried grass and their N fractions

All silages were of good quality, with similar DM concentration, *in vitro* organic matter digestibility and average metabolisable energy content of 10.5 (SD = 0.21) MJ/kg DM (Table 2). The pH and ammonia N concentration were lower for the formic acid treated silages than for untreated. The ^15^N-labelled silages and dried grass had higher CP and also higher SN concentration than the unlabelled forages (Table 3). On average, dried grass had a lower SN concentration than the silages. The CP concentration was higher in the silage SN fraction than in SN from dried grass, whereas the CP concentration was lower in the silage ISN and NDIN fractions than in the corresponding fractions in dried grass. The mean asymptotic gas production for dried grass and silages was 275 (SE = 6.5) and 281 (SE = 6.9) mL/g organic matter (OM), respectively. There were no significant differences between comparative substrates.

**Table 2:** Fermentation characteristics of silages (values expressed as g/kg DM, unless otherwise stated).

	FAS15^1^	UTS15	FAS	UTS
DM, g/kg	266	260	262	256
ME^2^	10.6	10.3	10.8	10.5
OMD^3^	725	708	735	720
pH	3.88	4.88	3.89	4.13
AN^4^, g/kg N	38	99	28	60
Lactic acid	86.4	49.1	84.3	73.7
Acetic acid	16.0	20.3	16.9	19.5
Propionic acid	3.6	11.4	5.1	2.9
Butyric acid	0.3	1.4	0.3	0.3
Ethanol	11.9	24.8	11.6	17.8

^1^ FAS15 and FAS = Formic acid-treated ^15^N-labelled and unlabelled grass silage, UTS15 and UTS = untreated ^15^N-labelled and unlabelled grass silage.

^2^ ME = metabolisable energy (MJ/kg DM).

^3^ OMD = *in vitro* OM digestibility.

^4^ AN = ammonia N concentration of silages and soluble N fractions.

**Table 3:** Chemical composition of forages and N fractions used *in vitro*.

	DM^2^, g/kg	g/kg DM			g/kg N		^15^N A% / N^4^
Item^1^		Ash	CP	NDF	Soluble N	AN^3^	
FAS15	860	66	155	521	772		1.642
FAS15-SN	974	NA	270	NA		90.0	1.645
FAS15-ISN	907	21	63	744			1.646
FAS15-NDIN	904	10	29	971			1.596
UTS15	883	71	166	550	785		1.563
UTS15-SN	971	NA	290	NA		123	1.593
UTS15-ISN	904	20	65	785			1.476
UTS15-NDIN	905	10	22	978			1.432
FAS	862	66	149	518	692		0.374
FAS-SN	973	NA	299	NA		95.9	0.371
FAS-ISN	908	21	70	740			0.371
FAS-NDIN	901	9	32	975			0.372
UTS	873	65	142	543	637		0.373
UTS-SN	969	NA	321	NA		115	0.374
UTS-ISN	905	18	72	776			0.374
UTS-NDIN	905	9	25	979			0.373
DG15	940	64	144	502	297		1.609
DG15-SN	954	NA	157	NA		14.3	1.691
DG15-ISN	906	28	139	714			1.607
DG15-NDIN	950	11	107	902			1.568
DG	938	59	132	500	237		0.369
DG-SN	954	NA	137	NA		15.4	0.372
DG-ISN	907	26	131	714			0.372
DG-NDIN	905	11	109	902			0.372

^1^ FAS15 and FAS = Formic acid-treated ^15^N-labelled and unlabelled grass silage, UTS15 and UTS = untreated ^15^N-labelled and unlabelled grass silage, DG15 and DG = ^15^N-labelled and unlabelled dried grass; SN = soluble N fraction; ISN = insoluble N fraction; NDIN = neutral detergent-insoluble N fraction.

^2^ DM content of feeds when used *in vitro*.

^3^ AN = ammonia N concentration of soluble N fractions.

^4 15^N A%/N = ^15^N-atom% of total N.

The N^15^ atom% excess of labelled forages was 1.27, 1.19 and 1.24 for the N^15^ labelled formic acid treated and untreated silage and dried grass, respectively. The N^15^ atom% excess in the SN fraction was slightly higher than that in whole forages, while it was about 3.5% lower in the NDIN fraction than in whole forages.

**Fig 2:**
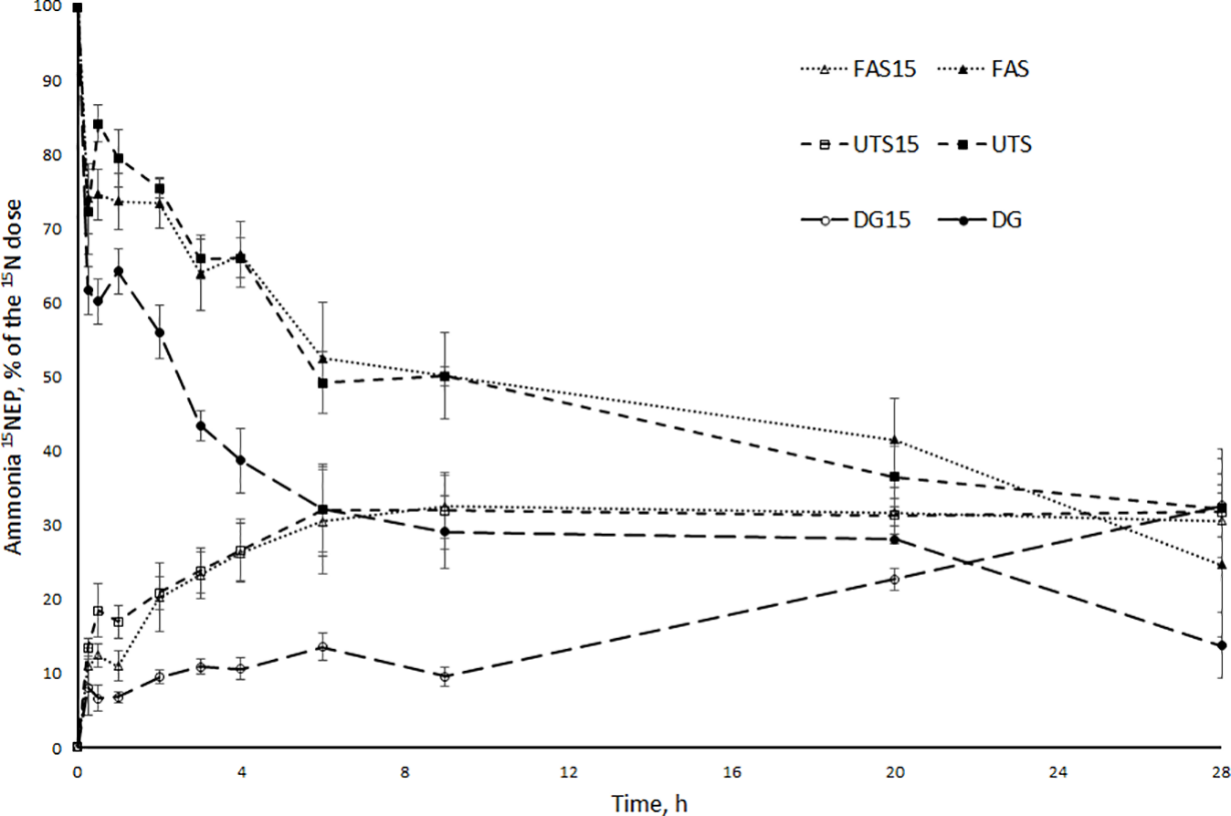
Changes in ammonia ^15^NEP sizes with forages. Ammonia ^15^N excess pool (^15^NEP) size (% of the initial dose) from ^15^N-labelled forages (open symbols) and ^15^N-labelled ammonia with unlabelled forages (filled symbols). Formic acid-treated silages (FAS15, FAS; dotted line), untreated silages (UTS15, UTS; dashed line), dried grasses (DG15, DG; long dashed line). Time × treatment interaction for ^15^N labelled forages *P* = 0.019 and unlabelled forages *P* = 0.002.

The appearance of ^15^N in the ammonia ^15^NEP from the N^15^ labelled untreated silage during the first 30 min was almost two-fold of that from N^15^ labelled formic acid treated silage and dried grass (Fig 2). After the first hour, the appearance rate of ^15^N from silages to ammonia ^15^NEP was almost identical until the end of the observation period (treatment × time interaction *P* = 0.019).

The initial ammonia ^15^NEP was calculated based on the ammonia N concentration and the extra N administered as (^15^NH_4_)_2_SO_4_ into incubation vessels. The disappearance of labelled ammonia ^15^N was rapid for all forages, with 25–38% of the initial ^15^N dose (212 μg) disappearing within 15 min (Fig 2). However the initial disappearance was followed by an increase in ammonia ^15^NEP. This pattern of rapid initial disappearance of excess ammonia ^15^N followed by an increase in ammonia ^15^NEP size between 15 and 60 min of incubation was observed for all fractions and also with the N^15^ labelled blank. During the incubation, the decrease of ammonia ^15^NEP was faster and reached a higher extent with dried grass than with silages (treatment × time interaction *P* = 0.002). After the initial changes the ammonia ^15^NEP in N^15^ labelled blanks, did not change significantly despite an increase in the total ammonia N pool size from 2.3 to 4.4 mg between 4 and 20 h.

### Soluble N

**Fig 3:**
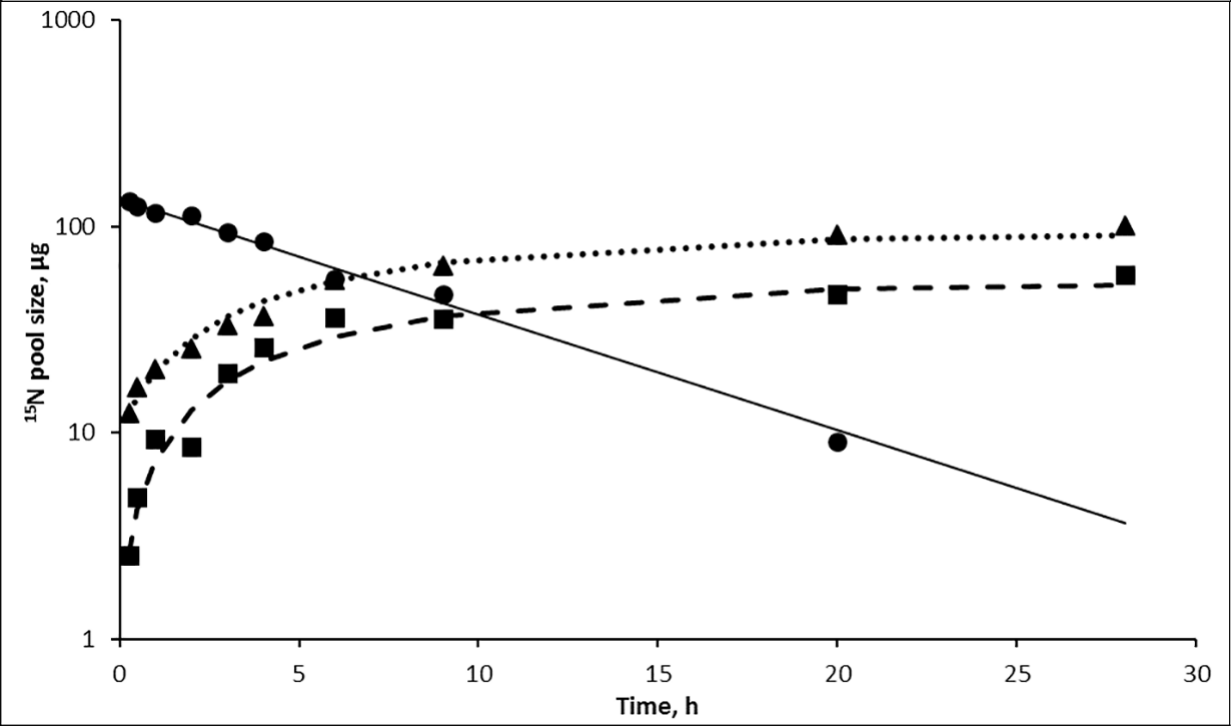
Predicted and observed ^15^NEP sizes with ^15^N-labelled SN fraction of FAS15-SN. Predicted (solid line) and observed (●) pool size of soluble non-ammonia ^15^N pool. Predicted (dotted line) and observed (▲) pool size of ammonia ^15^N pool. Predicted (dashed line) and observed (■) pool size of microbial ^15^N pool.

The SN in silages and dried grass contained 9.0–12.4% and 1.4–1.5% of ammonia N, respectively (Table 3). The excess of ^15^N in the labelled SN of dried grass substrate was only 51 μg, compared with 147 and 140 μg in the SN of formic acid treated and untreated silage substrates, respectively. Fig 3 presents the observed values and the model estimates for *in vitro* metabolism of only the SN fraction of labelled formic acid treated silage as an example, since the figures for the labelled untreated silage and dried grass SN fractions were similar. The first observation at 15 min indicated that 8, 18 and 11% of labelled N from the SN fraction in formic acid treated and untreated silage and dried grass, respectively, appeared in the ammonia N pool. By the end of the incubation period, about 67, 62 and 19%, respectively, of ^15^N in the initial SN fraction was recovered as ammonia N with minimal excess ^15^N remaining in the SNAN pool. The immediate microbial uptake of SN was 20% of the ^15^N dose in the SN fraction of labelled dried grass, but for the silages the predicted initial microbial uptake of SN was only 0.6–1.2%. The fractional degradation rate of the SN fractions of N^15^ labelled dried grass during the first 9 h were significantly (*P* = 0.016) faster (0.198 /h) than of the silages and lowest for the untreated silage (0.145 and 0.110 /h respectively).

The ^15^N enrichment of ammonia N pool increased linearly (R^2^ = 0.93 and 0.98) during the first 6 hours, with a rate of 0.148 and 0.235 /h for the ^15^N-labelled silage and dried grass SN substrates, respectively. However, apart from the initial decrease in ammonia ^15^NEP during 0–15 min, there were no significant changes during the first 9 hours when ^15^N was administered as ammonia N to unlabelled silage-SN fraction of unlabelled silage. For the SN of dried grass, the ammonia ^15^NEP decreased linearly (R^2^ = 0.96) from 121 to 94 μg (from 0.58 to 0.44 of initial dose) between 15 min and 9 hours during the incubation, but remained unchanged for the rest of the incubation period.

The rate of microbial uptake of SNAN estimated by the SN model was slightly faster for the SN fraction of labelled dried grass than for the SN fractions of silages (Table 4). The rate of SNAN degradation to ammonia N was fastest for the SN of labelled formic acid silage and slowest for the SN of labelled dried grass. However, the rate of ammonia N uptake was 4- to 9-fold higher with SN of dried grass than with SN of silages. The proportion of SN estimated by the SN model to be recovered immediately in the ammonia N pool was similar to the proportion of ammonia N in the SN fraction (Tables 3 and 4). The immediate uptake of SNAN and ammonia N by microbes was similar for both silages, but higher for the SN fraction of labelled dried grass. No recycling of microbial N to ammonia N was detected, based on the modelling of appearance of ^15^N in ammonia ^15^NEP.

**Table 4:** Parameter estimates for the soluble nitrogen (SN) model.

Parameter	FAS15-SN^1^		UTS15-SN		DG15-SN	
	Estimate	FSD^2^	Estimate	FSD	Estimate	FSD
kd_SNAN-AN^3^, /h	0.079	0.052	0.064	0.106	0.055	0.155
ks_SNAN-MN^4^, /h	0.050	0.057	0.047	0.080	0.057	0.161
P_1_^5^	0.065	0.102		0.172	0.073		0.137	0.066
P_2_^6^	0.007	0.291	0.013	0.249	0.198	0.064
	FAS-SN		UTS-SN		DG-SN	
ks_AN-MN^7^, /h	0.013	1.200	0.029	0.417	0.114	0.168
P_3_^8^	0.247	0.055	0.220	0.065	0.416	0.031

^1^ FAS15-SN = soluble N fraction (SN) of formic acid treated ^15^N labelled grass silage, UTS15-SN = SN of untreated ^15^N labelled grass silage, DG15-SN = SN of ^15^N labelled dried grass, FAS-SN = SN of unlabelled formic acid treated silage, UTS-SN = SN of unlabelled untreated grass silage, DG-SN = SN of unlabelled dried grass.

^2^ FSD = fractional standard deviation (SD / mean)

^3^ kd_SNAN-AN = rate of soluble non-ammonia N (SNAN degradation to ammonia N by microbes

^4^ ks_SNAN-MN = rate of direct microbial N synthesis from SNAN

^5^ P_1_ = proportion of SN immediately found in ammonia N pool

^6^ P_2_ = proportion of SN immediately found in microbial N pool

^7^ ks_AN-MN = rate of microbial N synthesis from ammonia N

^8^ P_3_ = proportion of ammonia N immediately taken up by microbes.

### Insoluble N

**Fig 4:**
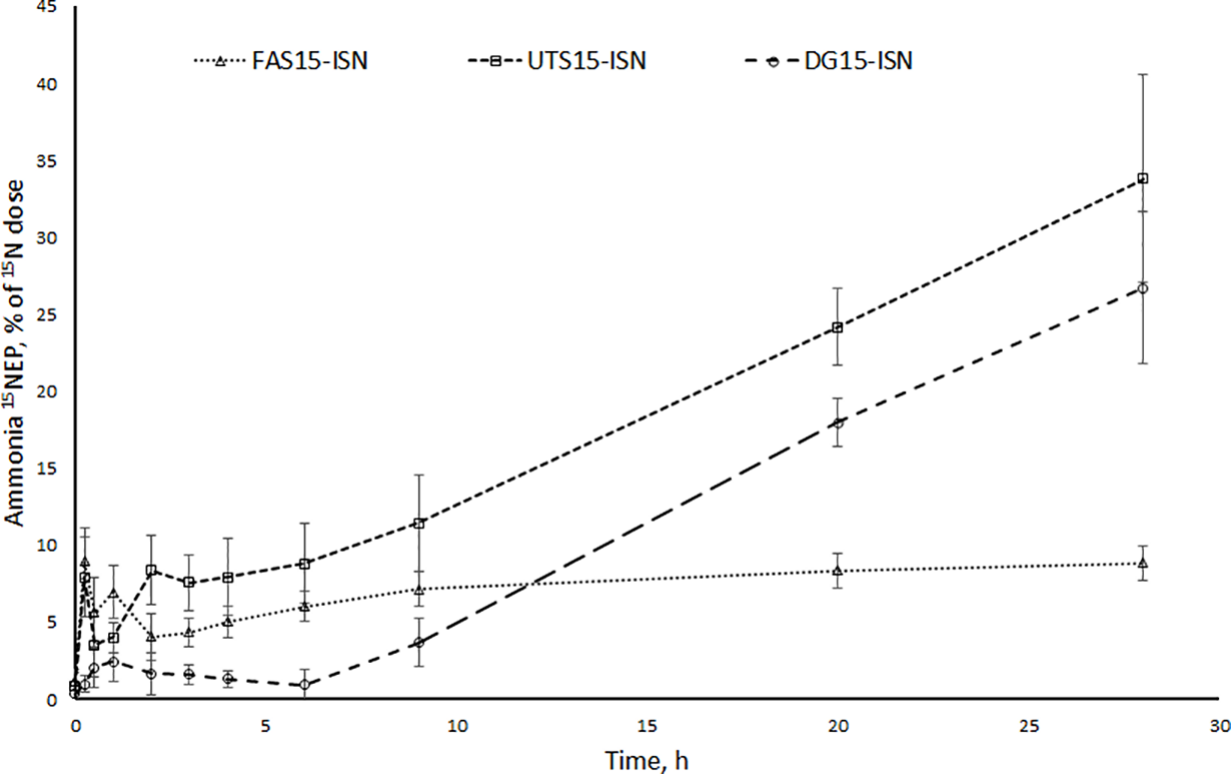
Changes in ammonia ^15^NEP sizes with ^15^N-labelled grass forage ISN fractions. Ammonia ^15^N excess pool (^15^NEP) size (% of the initial dose) Formic acid-treated silages (FAS15-ISN; dotted line), untreated silages (UTS15-ISN; dashed line), and dried grass (DG15-ISN; long dashed line). Time × treatment interaction is *P* < 0.001.

The first sampling at 15 min showed that 7.9 and 8.9% of the initial dose of ^15^N from the ISN fraction of labelled formic acid treated and untreated silage was recovered in ammonia ^15^NEP (Fig 4). With the ISN fraction of labelled dried grass, only 2.0% of the initial dose was recovered in ammonia ^15^NEP after 15 min of incubation, which remained virtually unchanged for the first 9 h. The percentage of initial ^15^N from labelled ISN fraction found in ammonia ^15^NEP after 28 hours was lower for the labelled formic acid treated silage than for the untreated silage and dried grass (14, 30 and 24%, respectively; *P* < 0.001).

**Fig 5:**
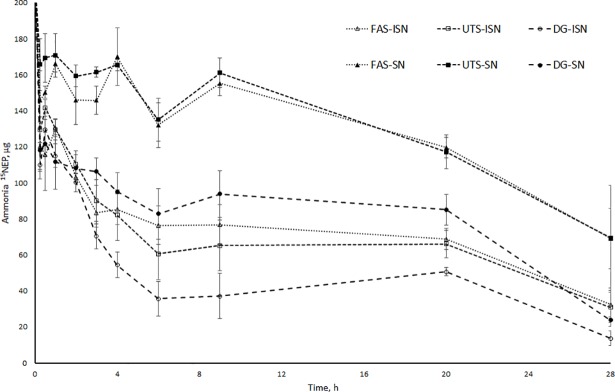
Changes in ammonia ^15^NEP size when ^15^N was administered into ammonia N. Ammonia ^15^N excess pool (^15^NEP) size (μg) with unlabelled soluble N (SN, filled symbols) and insoluble N (ISN, open symbols) fractions. Formic acid-treated silages (FAS; dotted line), untreated silages (UTS; dashed line), dried grass (DG15; long dashed line). Time × treatment interaction for ISN fraction is *P* = 0.14 and for SN fraction *P* < 0.001.

The rapid disappearance of ^15^N dose from the ammonia N pool (40–47% within 15 min) was not possible to model and therefore the initial condition of ammonia ^15^NEP was used as an adjustable parameter in the model. The ammonia ^15^NEP decreased linearly until 6 hours, after which there were no significant changes (Fig 5). The rate of microbial N synthesis from ammonia N was 1.6- and 2.3-fold higher for the ISN of untreated silage and dried grass compared with ISN of formic acid treated silage (Table 5). However, the rate of microbial N recycling to ammonia N was similar for all three forages.

**Table 5:** Parameter estimates for the insoluble nitrogen (ISN) model.

Parameter	FAS-ISN^1^		UTS-ISN		DG-ISN	
	Estimate	FSD^2^	Estimate	FSD	Estimate	FSD
ks_AN-MN^3^, /h	0.152	0.126	0.241	0.153	0.350	0.123
kr_MN-AN^4^, /h	0.072	0.165	0.084	0.215	0.072	0.182
P_1_^5^	0.355	0.041	0.295	0.093	0.314	0.098

^1^ FAS-SN = insoluble N fraction (ISN) of formic acid-treated unlabelled grass silage, UTS-ISN = ISN of untreated unlabelled grass silage, DG-ISN = SN of unlabelled dried grass.

^2^ FSD = fractional standard deviation (SD / mean).

^3^ ks_AN-MN = rate of microbial N synthesis from ammonia N.

^4^ kr_MN-AN = rate of microbial N recycling to ammonia N.

^5^ P_1_ = proportion of ammonia N immediately taken up by microbes.

The ^15^N disappearance from labelled ammonia ^15^NEP was faster and reached a higher extent with ISN fractions than with SN fractions (Fig 5). The decrease in ammonia ^15^NEP size was higher with the SN and ISN fractions of dried grass than with the corresponding silage N fractions. The decrease in ammonia ^15^NEP was greatest with the ISN fraction of dried grass, with 76% of the ammonia ^15^N disappearing in 9 hours, compared with 60% for the SN fraction of dried grass.

### Neutral detergent insoluble N

None of the ^15^N from the labelled silage NDIN fraction appeared in ammonia ^15^NEP and only 12% of ^15^N in the NDIN fraction of labelled dried grass was recovered as ammonia ^15^N by the end of the incubation.

The disappearance of ^15^N from the labelled ammonia ^15^NEP followed the same pattern as for ISN, but the differences between forages were smaller. The ammonia ^15^NEP decreased linearly (R^2^ = 0.94–0.98) until 6 hours, after which there were no significant changes during the rest of the incubation period.

## Discussion

### Forage quality and N fractions

The first objective of this study was to grow ^15^N-labelled and unlabelled timothy grass in as similar a way as possible, in order to achieve similar N fractionation in the forages for comparison of the effects of preservation method. The similar DM concentration in all silages (261 g/kg DM, SD = 0.4) and similar fermentation quality for corresponding ^15^N-labelled and unlabelled silages (Table 2) show that this objective was generally achieved. The effect of the acid-based additive in reducing silage pH and ammonia N concentration was as expected [29]. The higher CP concentration in the NDIN fraction of formic acid-treated silages than that of untreated silages may indicate reduced hydrolysis of fibre-bound N in treated silages [30]. The higher CP concentration in the dried grass NDIN fraction supports this assumption, as less breakdown/hydrolysis occur during drying than during ensiling [9]. Nsereko and Rooke [31] reported a higher proportion of peptides in soluble N in formic acid-treated silage than in untreated silage.

There were no significant differences in ^15^N enrichment between dried grass and different silages, but the lower enrichment of NDIN fraction and slightly higher enrichment of SN fraction compared with total N in the whole forages agree with the findings by Hristov *et al*. [17]. They reported much lower ^15^N enrichment in ADF-bound N than in other N fractions.

Pre-incubation of ruminal fluid proved effective in normalising the conditions between the runs. The average ammonia concentration between runs at the start of the incubation was 44.5 mg/L (SD = 2.1). Based on authors earlier experience [32] and with current data it is recommended that *in vitro* medium should always be pre-incubated when evaluating protein digestion. The extent and rate of gas production determined in this study suggests that the *in vitro* fermentation proceeded normally [33].

The rapid initial disappearance of ^15^N from labelled ammonia ^15^NEP observed for all samples remains unexplained. The proportional changes in the total ammonia N pool between the start and 15 min of incubation were not large enough to explain the decrease in ammonia ^15^NEP size. It can be speculated that this disappearance was due to N uptake into microbial cells, because the rumen fluid was deprived of ammonia during the pre-incubation. Blake *et al*. [34] observed an almost 50% decrease in ammonia ^15^N atom% excess in just 30 min after infusion of ^15^NH_4_Cl into the rumen of steers. In addition, they observed a significant increase in ^15^N enrichment of intracellular ammonia in bacteria after only 2 min, demonstrating the ability of microbes for rapid uptake of ammonia N. In such a short time, ammonia N cannot be completely used for microbial protein synthesis, which suggests that some of ammonia is taken into intracellular pools [35]. A substantial proportion of the ^15^N had reappeared in the ammonia ^15^NEP after 30 or 60 min in the present study, suggesting release of some of the absorbed ^15^N from microbes. This is supported by the slower ^15^N enrichment of bacterial AA than of intracellular ammonia reported by Blake *et al*. [34]. Previous tests conducted without pre-incubation of rumen fluid in our laboratory [unpublished data) indicated that microbial uptake and release of ammonia can occur in a short time and exceed the extent observed in the present study.

The increase in total ammonia production in the blanks observed after 20 hours may be due to microbial lysis [36]. However, since there was no increase in ammonia ^15^NEP, the increase in the ammonia N pool size mostly likely resulted from the lysis of protozoal or inoculum bacteria. Protozoa grow slowly and survive only in small numbers in this type of batch culture *in vitro* system [37], and therefore incorporation of ^15^N into protozoal cells was likely to be negligible.

### Soluble N

As expected, the ammonia N concentration in silage SN was higher than that in dried grass SN, since the ammonia concentration in hay is normally very low compared with that in silage [17,38]. The SNAN model fitted both the dried grass and silage data well, although random variation was greater for the dried grass data, likely due to lower N concentration in SN fractions of dried grasses compared with silage.

The greater proportion of the ^15^N in SN fraction of labelled untreated silage appeared to ammonia N pool during the first 15 min of incubation compared with the SN fraction of formic acid treated silage and dried grass (0.18, 0.08 and 0.11, respectively) may partly be because of the higher ammonia N concentration in SN fraction of untreated silage than in formic acid treated silage (Table 3). However, this does not explain the relatively high value with the SN fraction of labelled dried grass since the ammonia concentration in SN fraction of dried grass was only 10–15% of that in silage SN. These differences in degradation could be explained by differences in the rate of hydrolysis of different soluble proteins and to more favourable composition of SN in restrictedly fermented silage and dried grass [38,39,40]. Wallace [41] found that the protein adsorption rate to bacteria corresponded closely to their initial rate of hydrolysis. Soluble N in untreated grass silage has a significantly lower peptide concentration but greater free amino acids (**AA**) concentration compared with formic acid-treated grass silage [33,42]. Microbes may prefer peptides and AA from restrictedly fermented silage and use these components directly as N source for microbial synthesis [43,44]. This is also supported by the lower initial microbial uptake of ammonia ^15^N from labelled rumen fluid observed with whole silage and silage SN fractions than with the dried grass and SN of dried grass. The overall slower appearance of ^15^N into ammonia ^15^NEP and higher disappearance of ^15^N from labelled ammonia ^15^NEP with dried grass than silages suggest slower degradation [45, 46] and/or higher microbial N synthesis from direct incorporation of SN with dried grass [41].

The fractional degradation rates of SN fractions observed here were about two-fold lower than previously reported by Peltekova and Broderick [47]. In dairy cows, given a single dose of ^15^N-labelled grass silage SN fraction, the SNAN disappearance rate was 1.24 /h, with the rate of SNAN degradation to ammonia N being about five-fold faster than direct bacterial uptake of SNAN [48]. In our study the degradation rate of silage SN fractions to ammonia N was only about 50% faster than the rate of microbial uptake of SN, and with SN from dried grass the rates of uptake and degradation were similar. However, part of the difference can be due to microbes using some of the ammonia ^15^N produced from SN degradation for microbial N synthesis. This is supported by the fact that estimated rate of microbial N synthesis from ammonia N was slowest for the SN faction of formic acid treated silage (0.013 /h) and fastest for the SN fraction of dried grass (0.114 /h).

### Insoluble N

The ^15^N from silage ISN fractions recovered in ammonia ^15^NEP during the early stage of incubation suggests better availability of silage ISN for microbial synthesis than dried grass ISN [47]. This also may explain the lower ammonia ^15^NEP with the ISN fraction of the labelled formic acid treated silage than with the ISN fraction of the untreated silage at the end of incubation, indicating better utilisation of ISN from restrictively fermented silage.

Lower appearance of ^15^N in ammonia ^15^NEP from the ISN fraction of the labelled formic acid treated silage and the corresponding lower rate of microbial uptake of labelled ammonia ^15^N with the ISN of the unlabelled formic acid treated silage compared with untreated silage and dried grass suggests better utilisation of the ISN fraction of the formic acid-treated silage. In extensively fermented silage, the remaining N may be less available for microbial synthesis [4]. On the other hand, hay usually has lower degradability than silage when determined by the *in situ* method [10,49].

The greater extent of ^15^N uptake from labelled ammonia ^15^NEP with ISN fractions compared with SN fractions suggest that ammonia played a more important role as N source for microbial protein synthesis with the ISN fraction. These results are in agreement with observations by Peltekova and Broderick [47], who used ^15^N-labelled ammonia N to measure *in vitro* ruminal degradation of soluble and insoluble fractions of alfalfa silage and hay and observed lower bacterial ^15^N enrichment (0.474 N^15^ atom% excess) with soluble N fraction compared with the insoluble fraction (0.664 N^15^ atom% excess).

### Neutral detergent insoluble N

None of the dose of ^15^N in NDIN appeared in ammonia ^15^NEP. If any of the NDIN was degraded to ammonia N, this must have occurred intracellularly and the ammonia N must have been directly incorporated into microbial protein. This agrees with the suggestion that fibre-bound bacteria use protein directly without degrading it to the external ammonia N pool [50,51]. Cellulolytic bacteria have a preference for ammonia as N source [52], although they can also utilise other N sources [51,53]. Because of low CP concentration in NDF, particle-associated bacteria utilise degradation products directly for cell synthesis rather than excreting them into extracellular pools. In addition, the *in vivo* ruminal degradability of the NDIN fraction is rather low [54].

With both the unlabelled NDIN and ISN fractions, the ammonia ^15^NEP decreased only for the first 6–9 hours, after which it remained mostly unchanged or even increased. This observation supports the idea that rate of microbial lysis or recycling and ammonia uptake reached an equilibrium after 6 hours, as speculated previously concerning ammonia ^15^NEP changes in N^15^ labelled blanks. Wells and Russell [36] discussed bacterial lysis and presented similar data showing two-phasic ammonia ^15^N dilution kinetics for intraruminal enrichment of N following a single injection of ^15^N. Using measured data reported by Nolan and Leng [55], Wells and Russell [36] developed hypothetical values showing the dilution rate of ^15^N-labelled ammonia to be 14-fold greater in the first 5–6 hours than at later times. Thus it was concluded that about 35% or more ammonia N was turned over, prolonging the dilution of ^15^N-labelled ammonia.

### Implications

Using ^15^N labelled feed N fractions allows to study ruminal N metabolism in more detail both *in vitro* and *in vivo* [17]. The results have indicated that the degradation rate of SNAN fraction to ammonia N is not infinite and that quantitatively an important fraction escapes ruminal degradation. This is contrast to the assumptions of most feed protein evaluation systems in calculating RUP and MP. The evidence also suggest that SNAN is a better N source for rumen microbes than ammonia N. Therefore, feed protein evaluation systems could be improved by taking into account escape of the SNAN and the nature of N on microbial protein synthesis. Although rather labour-intensive, kinetic studies using ^15^N labelled feed N fractions could be used to determine parameter values required in both mechanistic and static feed evaluation models [48].

## Conclusions

Timothy grass forages labelled with ^15^N and preserved as formic acid-treated or untreated silage or as dried grass were used to estimate the ruminal degradation kinetics of different forage protein fractions *in vitro*. Formic acid treatment decreased ammonia N concentration in silage and reduced degradation and improved microbial N synthesis from ammonia N, but there were no significant differences in overall degradation of formic acid treated silages compared with untreated silage. Differences in degradation of SN fractions between forages indicated significantly higher true protein concentration in dried grass than in silages. The degradation rates of SN fractions were lower than normally observed *in vivo*. The microbial uptake of rumen ammonia was significantly greater with insoluble N fractions than with soluble N fractions, indicating higher importance of soluble N, compared to ammonia N for direct microbial synthesis. More work is needed to relate the results of *in vitro* predictions to *in vivo* conditions. The method of using ^15^N-labelled plants to study ruminal degradation kinetics is a promising approach for future *in vitro* and *in vivo* studies.

## Supporting information

S1 Table Supporting information raw data.xlsx. (XLSX)
